# Slowing of EEG Background Activity in Parkinson’s and Alzheimer’s Disease with Early Cognitive Dysfunction

**DOI:** 10.3389/fnagi.2014.00314

**Published:** 2014-11-18

**Authors:** Nina Benz, Florian Hatz, Habib Bousleiman, Michael M. Ehrensperger, Ute Gschwandtner, Martin Hardmeier, Stephan Ruegg, Christian Schindler, Ronan Zimmermann, Andreas Urs Monsch, Peter Fuhr

**Affiliations:** ^1^Department of Neurology, Hospitals of University of Basel, Basel, Switzerland; ^2^Swiss Tropical and Public Health Institute, University of Basel, Basel, Switzerland; ^3^Memory Clinic, University Center for Medicine of Aging Basel, Felix Platter Hospital, Basel, Switzerland

**Keywords:** Parkinson’s disease, Alzheimer’s disease, mild cognitive impairment, dementia, quantitative electroencephalography, neuropsychology

## Abstract

**Background:** Slowing of the electroencephalogram (EEG) is frequent in Parkinson’s (PD) and Alzheimer’s disease (AD) and correlates with cognitive decline. As overlap pathology plays a role in the pathogenesis of dementia, it is likely that demented patients in PD show similar physiological alterations as in AD.

**Objective:** To analyze distinctive quantitative EEG characteristics in early cognitive dysfunction in PD and AD.

**Methods:** Forty patients (20 PD- and 20 AD patients with early cognitive impairment) and 20 normal controls (NC) were matched for gender, age, and education. Resting state EEG was recorded from 256 electrodes. Relative power spectra, median frequency (4–14 Hz), and neuropsychological outcome were compared between groups.

**Results:** Relative theta power in left temporal region and median frequency separated the three groups significantly (*p* = 0.002 and *p* < 0.001). Relative theta power was increased and median frequency reduced in patients with both diseases compared to NC. Median frequency was higher in AD than in PD and classified groups significantly (*p* = 0.02).

**Conclusion:** Increase of theta power in the left temporal region and a reduction of median frequency were associated with presence of AD or PD. PD patients are characterized by a pronounced slowing as compared to AD patients. Therefore, in both disorders EEG slowing might be a useful biomarker for beginning cognitive decline.

## Introduction

Mild cognitive impairment (MCI) is a prodromal syndrome of neurodegenerative dementia without significant impairment in activities of daily living (ADL); however, it is unspecific as it can be caused by various pathologies (Winblad et al., 2004), and a considerable part of MCI patients remain stable or improve over time (Ritchie et al., 2001). Diagnostic criteria for MCI due to Parkinson’s disease (PD-MCI) and Alzheimer’s disease (AD-MCI) were established (Albert et al., 2011; Litvan et al., 2012). The progression rate in PD-MCI to PD dementia (PDD) is approximately 60% over 4 years (Janvin et al., 2006); the rate of conversion to AD in MCI patients varies depending on study design and definition of MCI, though maximally one to two fifths of MCI patients with amnestic impairments at baseline progress to dementia within 2–3 years of follow-up (Schmidtke and Hermeneit, 2008; Duara et al., 2011). However, novel treatment strategies require initiation of treatment at the earliest possible time (Panza et al., 2012), and, therefore, the corroboration of the diagnosis and the identification of the cause of MCI is very important.

Quantitative electroencephalography (qEEG) has increasingly been used to characterize cognitive impairment in different disorders (Fonseca et al., 2009; Roh et al., 2011). The eyes closed resting state qEEG of patients suffering from dementia due to AD is characterized by a shift to lower frequencies (Penttilä et al., 1985; Duffy et al., 1995; Bennys et al., 2001; Czigler et al., 2008). The same observation is reported in PD patients with cognitive decline (Caviness et al., 2007; Fonseca et al., 2009; Bousleiman et al., 2014). In contrast to AD, slowing of electroencephalogram (EEG) can already be found in *de novo* PD patients without any cognitive deficits (Stoffers et al., 2007). Comparison of AD and PDD patients with a similar degree of overt dementia showed more pronounced EEG slowing in PDD (Babiloni et al., 2011; Fonseca et al., 2013). However, it is unknown whether qEEG measures reliably discriminate between the two diseases at the beginning of the dementing process or whether they simply correlate with the extent of cognitive decline. Thus, the present study aims at characterizing qEEG parameters in PD-MCI and AD-MCI.

## Materials and Methods

### Patients

Twenty patients with PD and cognitive decline (see Table 1) were recruited from the outpatient clinic for movement disorders of the University Hospital of Basel. PD was diagnosed according to UK Parkinson’s disease brain bank criteria. The diagnosis of PD-MCI (*N* = 15) was based on Litvan et al. (2012). Diagnosis of “probable PDD” (*N* = 5) was made according to Emre et al. (2007), p. 007. All PD patients were treated with dopaminergic drugs [median levodopa-equivalent dose (LED) = 829 mg; range 188–3044 mg].

**Table 1:** **Demographic characteristics and medications**.

	NC	AD^a^	PD^a^	*p*-Value^a^	Sub-group comparisons^b^
Age (years)	73.5 (67–83)	73.5 (57–87)	74 (60–84)	0.77	
Education (years)	13.5 (10–19)	14 (8–20)	15.5 (8–20)	0.60	
Gender (F/M)	11/9	10/10	5/15	0.12	
MMSE	29 (28–30)	28 (24–30)	29 (24–30)	0.02*	NC > AD*, NC > PD*, AD = PD
MMSE and CDT	9 (7–9)	9 (5–9)	8.5 (4–9)	0.34	
l-DOPA	–	–	18/20		
DOPA-agonists	–	–	13/20		
MAO-inhibitors	–	–	6/20		
Amantadine	–	–	2/20		
COMT-inhibitor	–	–	2/20		
AChE-inhibitors	–	1/20	2/20		
Antidepressants	–	3/20	4/20		
Neuroleptics	–	–	1/20		
Benzodiazepines	1/20	1/20	1/20		

*^a^*p*-Values for ANOVA, Kruskal–Wallis test*.

*^b^Mann–Whitney *U* test*.

*p < 0.05, “ =” no significant difference.

Thirty-seven outpatients (Table 1) with either amnestic MCI (AD-MCI, *N* = 12) or mild dementia due to probable AD (*N* = 8) were recruited from the Memory Clinic, University Center for Medicine of Aging, Basel. Thereof 20 patients were matched to the PD group for gender, age, and education. MCI due to probable AD was diagnosed according to Winblad et al. (2004). Dementia due to probable AD was diagnosed according to McKhann et al. (1984).

Exclusion criteria consisted of MMSE score (Folstein et al., 1975) <24/30, significant psychiatric or organic brain disorders other than PD or AD, any other severe illness, and drug treatment influencing EEG recordings (antiepileptic or antipsychotic drugs).

Twenty normal controls (NC) were matched to both patient groups according to gender, age, and education (Table 1). Inclusion criteria were a subjective report of good health and a neuropsychological examination within normal limits. Exclusion criteria were a past and/or current diagnosis of any major brain disorder, alcoholism, psychiatric disorder, general anesthesia within the last 3 months and cognitive problems. To compare global cognitive function between both patient groups, we chose a combination of the MMSE and the clock drawing test (CDT) (Thalmann et al., 2002). The study protocol was approved by the local ethics committee. Written informed consent was provided by all participants.

### Neuropsychology

Patients were examined with a comprehensive battery of neuropsychological tests [see Hatz et al. (2013) for details]. Raw scores of tests were transformed into demographically (age, gender, and education) adjusted *z*-scores (Berres et al., 2000). Cognitive performance was judged to be impaired when *z*-scores were less than −1.28, i.e., below the 10th percentile.

### EEG recording

Electroencephalogram was recorded with a 256-channel EEG System (Netstation 300, EGI, Inc., Eugene, OR 97403, USA; DC-amplifier; sampling rate: 1000 Hz; high-pass filter: 0.01 Hz; vertex-reference, impedance ≤40 kΩ). They were instructed to relax, but to stay awake and to minimize eye and body movements. A continuous EEG with closed eyes was recorded for 12 min. During data acquisition, a subset of electrodes was monitored online by a technician to check for vigilance and artifacts. For patients taking benzodiazepines for sleep deprivation (one patient per group), medication was discontinued before EEG recording for at least 48 h. Only benzodiazepines with short half-life were allowed. One patient of the PD group was taking low-dose quetiapine for sleep deprivation, three patients were taking acetylcholinesterase inhibitors.

### Processing of EEG data

Two minutes of EEG data (single segments of at least 40 s) without artifacts or signs of sleep and drowsiness were visually selected, filtered (0.5–70 Hz, 2400 order least-squares filter) and down-sampled (500 Hz). Data from 214 electrodes (excluding cheek and neck electrodes) were subjected to automated artifact detection (Hatz et al., 2014). Resulting EEG data were re-referenced to average reference and bad channels were interpolated with the spherical spline method. Power spectra were calculated from epochs of 4 s duration (Welch’s method, spectral resolution 0.25 Hz) using a 80% Hanning window. The relative power for each frequency band was computed as the ratio between the absolute bandpower and the bandpower from 1 to 30 Hz. Relative power in the delta- (1–4 Hz), theta- (4–8 Hz), alpha1- (8–10 Hz), alpha2- (10–13 Hz), and beta- (13–30 Hz) bands were logit-transformed [*t*(*x*) = log(*x*/(1 − *x*)] to achieve an approximate normal distribution (Gasser et al., 1982). Global band powers (average over all 214 electrodes) and regional band powers were computed. For regional analyses, electrodes were grouped into 10 regions of interest (ROI) corresponding to the frontal, central, temporal, parietal, and occipital areas bilaterally (Stoffers et al., 2007). Median frequency was calculated in a spectral window between 4 and 14 Hz at occipital electrodes. For calculation of the median frequency, a “center of gravity” approach was chosen, taking into account all spectra generated for a single subject.

### Statistics

Demographic characteristics were compared between groups using non-parametric tests. EEG variables and results of neuropsychological tests were compared between the three groups using ANOVA. Subsequently, *post hoc t*-tests between sub-groups were applied. In case of regional EEG power analysis, permutation tests (statistics: ANOVA, *t*-test, number of 10,000 permutations) were used (Nichols and Holmes, 2002). A logistic regression analysis with backwards elimination to classify both groups was performed using the significant EEG measures from permutation tests as independent variables. Age, education, and gender served as covariates. Subsequently, a receiver–operator-characteristic (ROC) analysis was performed. Results with *p*-values <0.05 were considered significant. Comparison of demographic characteristics, global relative band power, neuropsychological results between groups and regression, as well as ROC analysis were done using R^®^. Frequency analysis, ANOVA’s, *t*-tests, and permutation tests were performed with Matlab^®^.

## Results

### Demographics

No significant differences between the patient groups regarding age, education, the combined MMSE/CDT, and gender were found (Kruskal–Wallis test, see Table 1). MMSE scores of both patient groups were similar and significantly smaller than those of the NC group (*p* = 0.04).

### Neuropsychology

Table 2 gives results of group- and sub-group comparisons. Sub-group comparison found differences between PD and AD patients in alertness/reaction time and divided attention/errors and in verbal memory (encoding, recall, and recognition).

**Table 2:** **Results of the neuropsychological assessments [age-, gender, and education corrected *z*-values (Berres et al., 2000)]**.

	NC	AD^a^	PD^a^	*p*-Value^a^	Sub-group comparisons^b^
Alertness	−0.2 (−1.8 to 0.7)	−0.3 (−1.8 to 2.3)	0.1 (−2.1 to 2.1)	0.14	
Alertness RT	0.3 (−1.5 to 2.3)	−0.1 (−2.3 to 2.8)	−1.1 (−2.1 to 0.5)	0.004	NC = AD, NC > PD***, AD > PD*
Divided attention: errors	0.2 (−1.9 to 2.3)	0.2 (−1.2 to 2.3)	−1.5 (−2.1 to 2.3)	0.01	NC = AD, NC > PD*, AD > PD**
TMT-A	0.4 (−1.3 to 3.5)	0 (−2.6 to 3.5)	−0.3 (−3 to 1.1)	0.06	
Corsi blocks fw	0 (−1.2 to 1.2)	0 (−1.5 to 1.8)	−0.6 (−1.8 to 1.2)	0.32	
Digit span fw	0.1 (−2.9 to 1.8)	−0.5 (−2 to 2.1)	0.2 (−1.1 to 3)	0.10	
Corsi blocks bw	0.5 (−0.8 to 1.9)	−0.4 (−3.5 to 1.2)	−0.1 (−2.1 to 1.5)	0.01	NC > AD**, NC = PD, AD = PD
Digit span bw	0.3 (−1.7 to 2.3)	−0.5 (−1.7 to 2.9)	0 (−1.1 to 2.3)	0.41	
Figures fluency	−0.1 (−2 to 2)	−1 (−3.7 to 0.7)	−0.8 (−1.9 to 1.2)	0.01	NC > AD**, NC > PD*, AD = PD
Phonemic fluency	0 (−1.1 to 2.6)	−0.4 (−2.7 to 1.2)	−0.2 (−3.3 to 2)	0.23	
Semantic fluency	0.3 (−2.3 to 2)	−0.9 (−2.5 to 0.7)	−0.5 (−1.8 to 0.8)	0.002	NC > AD**, NC > PD*, AD = PD
Boston naming test	−0.4 (−1.9 to 1.7)	−0.4 (−2.3 to 1.3)	−0.6 (−3.3 to 1.3)	0.20	
Encoding	−0.1 (−1.7 to 1.8)	−2 (−3.8 to −0.7)	−1.3 (−3.7 to 0.2)	<0.001	NC > AD***, NC > PD***, PD > AD**
Recall	0.3 (−2.1 to 1.4)	−2.5 (−4 to −0.3)	−1.3 (−3.3 to 0.4)	<0.001	NC > AD***, NC > PD**, PD > AD**
Recognition	0.2 (−2.4 to 1.7)	−1.6 (−4.5 to 1.7)	−0.6 (−3 to 1.6)	<0.001	NC > AD***, PD > AD**, NC = PD

*^a^*p*-Values for Kruskal–Wallis test*.

*^b^*p*-Values for Mann–Whitney *U* test*.

*p < 0.05, **p < 0.01, ***p < 0.001, “ =” no significant difference.

### Quantitative EEG

Global relative theta and global relative alpha2 power differed between the three groups. In sub-group comparison, NC had lower theta power compared PD and tended to lower theta power compared to AD. AD tended to have lower global theta with respect to PD, which had lower power in the alpha2 band compared to NC. Detailed results are given in Table 3.

**Table 3:** **Global relative power and median frequencies (median and range)**.

	NC	AD^a^	PD^a^	*p*-value^a^	Sub-group comparisons^b^
Delta (1–4 Hz)	19.5 (6.1–40.7)	19.4 (7.5–32.9)	20.4 (7.6–37.0)	0.59	
Theta (4–8 Hz)	11.2 (5.7–61.6)	16.5 (6.5–46.9)	24.4 (9.4–56.6)	0.002	NC < AD^#^, NC < PD**, AD < PD*
Alpha1 (8–10 Hz)	13.7 (5.8–56.2)	25.7 (5.5–55.4)	16.9 (6.1–37.0)	0.39	
Alpha2 (10–13 Hz)	14.2 (2.7–56.1)	11.8 (4.4–36.2)	9.3 (4.1–25)	0.04	NC > PD**, AD = NC, AD = PD
Beta (13–30 Hz)	24.2 (11.4–42.1)	18.9 (7.5–48.2)	22.3 (8.8–28.3)	0.30	
Median frequency	9.2 (8.1–10.4)	8.8 (7.1–10.5)	8.1 (6.9–9.4)	<0.001	NC > AD*, NC > PD***, AD > PD*

*^a^*p*-Values for ANOVA*.

*^b^*p*-Values for *t*-tests*.

^#^*p* < 0.1, *p < 0.05, **p < 0.01, ***p < 0.001, “ =” no significant difference.

**Figure 1:**
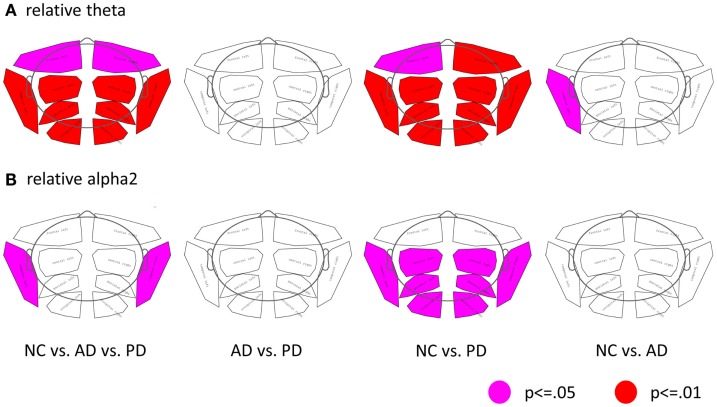
**Differences of regional band power between the three groups (NC, AD, and PD)**. **(A)** Relative theta power, **(B)** relative alpha2 power. (First column: ANOVA, second to forth columns: *t*-tests; pink: *p* ≤ 0.05; red: *p* ≤ 0.01; corrected for multiple comparison by permutation).

Regional analysis of relative power data revealed group and sub-group differences in the theta- and alpha2 bands as depicted in Figure 1. In both patient groups, the most significant differences as compared to NC were found in the left temporal region.

Occipital median frequency differed significantly between groups with the following pattern found in the *post hoc* analysis: NC > AD (*p* < 0.05), NC > PD (*p* < 0.001), and AD > PD (*p* < 0.05).

As the median frequency was significantly different between AD and PD, it was chosen as independent variable for a logistic regression model to classify the two groups. A significant classification of both groups (*p* = 0.02) was achieved. After backwards elimination, the resulting model included only median frequency as predictor (*p* = 0.03). ROC analysis yielded an AUC of 0.72 with a sensitivity and specificity at the best index of Youden (0.45) of 60 and 85%, respectively. The positive predictive and negative predictive values were 80 and 68%, respectively.

### Control analysis

We checked for a confounding influence of the variables MMSE, LED, alertness, age, and gender on the power values, by entering the variables into linear models with stepwise backwards elimination. No significant effects of the potentially confounding variables on the qEEG parameters were found. We conducted sensitivity analysis by running the statistics on all but one PD patient taking neuroleptics and on all but three patients taking AChE-inhibitors. The outcome was not affected as similar results and significances were obtained with and without those patients.

## Discussion

The main alteration of EEG in patients with subtle cognitive deficits due to AD or PD consists in an increase of relative theta power, predominantly in the temporal regions and in a slowing of the occipital median frequency. The slowing is more pronounced in PD than in AD patients.

Bi-temporal increase of relative theta power in demented PD and AD patients is a known finding (Babiloni et al., 2011). While in PD, EEG slowing occurs already before cognitive decline (Berendse and Stam, 2007), it is observed in AD with incipient MCI (Roh et al., 2011).

Dysfunction of the cholinergic system is common in both, AD and PD (Mesulam et al., 2004; Bohnen and Albin, 2011) and an overlap of histopathological (Hughes et al., 1993; Emre, 2003) and biochemical changes (Alves et al., 2014) is known. EEG frequency is accelerated by cholinergic function and responds to therapy with acetylcholinesterase inhibitors in AD und PD (Fogelson et al., 2003; Babiloni et al., 2013). This fact could explain partly the slowing of EEG in both patient groups in our sample. Interestingly, EEG slowing is more pronounced in PD than in AD in the present study. This is in line and extends the findings by Babiloni et al. who found similar results AD and PD patients with more advanced cognitive dysfunction This difference may reflect a greater cholinergic deficit in cognitively impaired patients with PD-compared to AD (Bohnen et al., 2003; Kotagal et al., 2012) and reflects the degeneration of the cholinergic system as an important factor for cognitive decline in PD (Emre et al., 2004).

The advantage of the present study is the comparison of demographically well balanced groups of patients with AD or PD at incipient cognitive decline with a group of NCs. Limitations include the relatively small sample sizes and the inherent failure of AD-MCI patients to formally fulfill the diagnostic criteria of AD. Longitudinal assessment of these patients is warranted.

## Conflict of Interest Statement

Nina Benz: none; Florian Hatz: none; Habib Bousleiman: none; Michael M. Ehrensperger: none; Ute Gschwandtner: Support from Mach-Gaensslen-Foundation, Gossweiler Foundation, Parkinson Schweiz, Synapsis Foundation, Botnar Foundation; Martin Hardmeier: none; Stephan Ruegg: Swiss National Science Foundation; Christian Schindler: Swiss National Science Foundation; Ronan Zimmermann: none; Andreas Urs Monsch: Swiss National Science Foundation, Parkinson Schweiz, Gossweiler Foundation, Synapsis Foundation, Novartis, Novartis Research Foundation; Peter Fuhr: Support of research from Swiss National Science Foundation, Mach-Gaensslen-Foundation, Gossweiler Foundation, Parkinson Schweiz, Synapsis Foundation, Botnar Foundation, Freiwillige Akademische Gesellschaft Basel, Novartis Research Foundation, Novartis, Roche, AbbVie.
